# Impact of High‐Fat Diet on Biochemical Changes Following Experimental Myocardial Infarction

**DOI:** 10.1111/jcmm.70984

**Published:** 2025-12-12

**Authors:** Katarina Bujnova, Andrej Barta, Jan Lakota, Martina Cebova

**Affiliations:** ^1^ Department of Neuro‐Cardiovascular Interaction, Institute of Normal and Pathological Physiology Centre of Experimental Medicine, Slovak Academy of Sciences Bratislava Slovakia; ^2^ Institute of Pathophysiology, Faculty of Medicine Comenius University Bratislava Slovakia

**Keywords:** high‐fat diet, myocardial infarction, nitric oxide, nuclear factor kappa B, toll‐like receptor

## Abstract

Myocardial infarction (MI) is a sudden necrosis of cardiomyocytes, often caused by atherosclerosis, with obesity being a significant risk factor. This study aimed to evaluate the effects of a high‐fat diet (HFD) on post‐MI myocardial remodelling, focusing on inflammatory signalling, nitric oxide synthase (NOS) dysregulation and oxidative stress. Nine‐week‐old Wistar Kyoto rats fed a control diet or a HFD for 4 weeks, followed by 20 min of left descending coronary artery occlusion and 7 days of reperfusion. Plasma levels of the proinflammatory cytokines TNF‐α and IL‐6 were measured using a Bioplex kit. NOS activity was assessed via [^3^H]‐l‐citrulline production, while the expression of eNOS, iNOS, NFκB and TLR4 in cardiac tissue was analysed by Western blot. Lipid peroxidation was assessed by measuring conjugated diene concentrations in cardiac tissue. MI and HFD both increased proinflammatory cytokine levels, reduced NOS activity and eNOS expression and increased iNOS expression. NFκB was more highly expressed after MI in control‐fed animals. Notably, TLR4 expression was reduced by HFD and remained unchanged post‐MI. Conjugated dienes were elevated post‐MI and further increased by HFD. These findings demonstrate that HFD exacerbates post‐MI inflammation and oxidative stress, impairing nitric oxide signalling and promoting adverse cardiac remodelling.

## 1 Introduction

Cardiovascular diseases are the main cause of death worldwide. Approximately 18 million people die annually due to cardiovascular diseases, of which up to 85% are myocardial infarctions and strokes [1]. Myocardial infarction (MI) is the sudden death of cardiomyocytes and is most often caused by atherosclerosis. Atherosclerotic plaque located in the coronary artery stops blood flow to the area of myocardium, causing damage to the heart muscle due to the lack of oxygen. Pathological processes characterised by dilatation and remodelling of the left ventricle after MI can lead to an increase in the remaining muscle mass and functional changes that involve both the ischemic and nonischemic zones of the myocardium [2].

Currently, we are facing an epidemic of obesity, the prevalence of which is increasing annually. By the year 2050, it is estimated that more than half of the global adult population, representing approximately 3.8 billion individuals, will be overweight or obese [3]. Obesity, an important risk factor for cardiovascular diseases, is associated with comorbidities such as hypertension, dyslipidemia, insulin resistance and hyperglycemia, which together are components of metabolic syndrome and contribute to the development of inflammatory vascular disease and thus to the occurrence of heart attack [4]. Obesity‐mediated changes are characterised by the release of proinflammatory and inflammatory cytokines as well as reactive oxygen species (ROS). This cytokine storm exacerbates the pathological cascade, potentially contributing to lipid peroxidation and subsequent cardiac remodelling, as demonstrated in various studies [5, 6]. Moreover, myocardial infarction triggers a robust inflammatory response characterised by the infiltration of immune cells and the generation of ROS, both of which contribute to the detrimental consequences observed after MI. Dying cardiomyocytes release damage‐associated molecular patterns (DAMPs), including Toll‐like receptor 4 (TLR4) or high mobility group box 1 (HMGB1) proteins. Nuclear factor kappa B (NFκB) can subsequently be activated. This activation triggers a signalling cascade leading to the upregulation of the expression of proinflammatory cytokine genes, including interleukin‐6 (IL‐6) and tumour necrosis factor‐α (TNF‐α), as demonstrated by Silvis et al. [7]. These proinflammatory cytokines subsequently promote the recruitment and activation of neutrophils and macrophages to the infarcted area [8]. Importantly, this inflammatory response can have a double‐edged sword effect. While it plays a crucial role in clearing damaged tissue and initiating repair processes, excessive or dysregulated inflammation can contribute to further tissue injury and worsen clinical outcomes following MI.

Nitric oxide (NO) is a signalling molecule with important functions in maintaining healthy blood vessels. This molecule has vasodilatory, anti‐inflammatory and antioxidant effects. However, the effect of NO on MI depends on its concentration and the nitric oxide synthase (NOS) isoform that produces it. NO formed by inducible NOS (iNOS) at high concentrations has a proatherogenic effect and contributes to the development of endothelial dysfunction when it competes with endothelial NOS (eNOS) for tetrahydrobiopterin (BH4), while eNOS uncouples and generates ROS. This molecule starts producing ROS instead of beneficial NO. Additionally, iNOS itself can generate harmful superoxide anions [9, 10]. Superoxide and NO are both highly reactive, and together, they form the powerful oxidant peroxynitrite, which causes apoptosis and cell necrosis, further enlarging the infarcted lesion. ROS are also involved in infarct‐induced tissue necrosis as well as in the progression of atherosclerosis [11]. Studies have shown that the activation of inflammatory molecules such as cytokines and iNOS significantly worsens myocardial injury. The excessive and sustained production of NO by iNOS can potentially contribute to myocardial damage through free radicals [12, 13, 14].

We hypothesise that the combined effect of myocardial infarction and high‐fat diet (HFD) feeding exacerbates cardiac inflammation, oxidative stress and fibrotic remodelling through dysregulation of nitric oxide synthase isoforms, upregulation of proinflammatory cytokines and altered activation of key signalling pathways including NFκB and TLR4. We further propose that HFD‐induced visceral adiposity may contribute to systemic inflammation and oxidative damage, thereby amplifying myocardial injury and remodelling independently of significant changes in blood pressure. The primary aim of this study was to elucidate the impact of a high‐fat diet on molecular and biochemical changes in the myocardium following myocardial infarction. Specifically, we sought to characterise the spatial distribution and extent of inflammatory responses and oxidative stress in different myocardial zones (infarct, injury and nonischemic) during the ischemia/reperfusion period. Furthermore, we aimed to investigate the interplay between dietary‐induced adiposity, inflammatory cytokine expression, NOS isoform regulation and ROS production, in order to better understand the mechanisms contributing to adverse post‐infarction cardiac remodelling under metabolic stress conditions. This study provides a novel integrative approach by establishing a link between HFD‐induced metabolic alterations and nitric oxide synthase dysregulation, inflammatory signalling and oxidative stress in the context of myocardial infarction. By examining the interplay between TLR4/NFκB signalling, NOS isoform expression and lipid peroxidation across different myocardial zones, new mechanistic insight is offered into how metabolic stress worsens post‐MI remodelling. Moreover, this work highlights potential molecular targets for therapeutic interventions aimed at mitigating obesity‐related cardiac injury and improving post‐infarction outcomes.

## 2 Materials and Methods

### 2.1 Chemicals

Most of the chemicals and reagents used were obtained from Sigma–Aldrich (Saint‐Louis, MO, USA); otherwise, the company is indicated.

### 2.2 Animals and Treatment

The experiments were carried out on Wistar Kyoto rats born in an accredited breeding establishment (SK UCH 03018) at the Institute of Normal and Pathological Physiology, Center of Experimental Medicine of the Slovak Academy of Sciences (INPP CEM SAS). All procedures and experimental protocols were performed in accordance with institutional guidelines and were approved by the State Veterinary and Food Administration of the Slovak Republic (Ro‐3587/19‐221) and by an ethical committee of the INPP CEM SAS according to the European Convention for the Protection of Vertebrate Animals used for Experimental and other Scientific Purposes, Directive 2010/63/EU of the European Parliament. The study was conducted and is reported in accordance with the ARRIVE guidelines (https://arriveguidelines.org), in line with the editorial policies of Molecular and Cellular Biochemistry.

All animals were weaned after 21 days. The animals were housed in groups of 2–4 animals in a room with a maintained temperature (22°C ± 2°C), relative humidity of 55%–65% and a 12‐h light/dark cycle. Nine‐week‐old male rats were randomly divided into four groups: (1) control sham‐operated WKY rats (CONTROL SHAM); (2) control WKY rats with experimentally induced MI (CONTROL MI); (3) sham‐operated WKY rats fed a high‐fat diet (HFD SHAM); and (4) WKY rats fed a high‐fat diet with experimentally induced MI (HFD MI) (*n* = 8 in each group). Until 9 weeks of age, all animals were fed a control diet (Ssniff R/M‐H phyt‐arm); thereafter, half of them were fed a high‐fat diet (Ssniff E15725). All animals had free access to their diet and drinking water. The composition of HFD is presented in Table 1. Blood pressure was measured noninvasively in prewarmed animals by tail‐cuff plethysmography (The IITC Life Science, MRBP blood pressure system) before surgery (week 12) and at the end of the experiment (week 13). Body weight (BW) was determined every week. At week 13, surgical intervention was performed. Seven days after surgery, the rats were sacrificed by decapitation, and the tissues were collected. At the end of the experiment, heart weight (HW), kidney weight (KW), retroperitoneal fat weight (RFW) and tibia length (TL) were measured. The relative heart weight was calculated as the HW/BW ratio and (HW/TL) ratio to evaluate the degree of cardiac hypertrophy. Plasma levels of TNF‐α and IL‐6 were determined using a commercial Bio‐Plex Pro Rat Cytokine Kit (Bio‐Rad, Hercules, CA, USA). Cardiac troponin T concentrations were measured with a Rat Cardiac Troponin T SimpleStep ELISA Kit (Abcam, Cambridge, UK). Catalase activity was assessed using a Catalase Activity Assay Kit (Abcam, Cambridge, UK), and superoxide dismutase (SOD) activity was determined with a SOD Activity Assay Kit (Sigma‐Aldrich, St. Louis, MO, USA); results are expressed as U/mL for plasma, according to the manufacturers' instructions.

**TABLE 1:** Composition of the high‐fat diet.

Crude nutrients	Control (%)	HFD (%)
Crude protein (*N* × 6.25)	19.2	20.2
Crude fat	4.1	35.1
Crude fibre	6.1	—
Crude ash	6.9	4.6
Starch	38.2	0.1
Sugar	—	17.0
Dextrins	19.4	19.2
*N* free extracts	60.4	37.6

### 2.3 Experimentally Induced Myocardial Infarction

Experimental MI was induced in 12‐week‐old rats by ligation of the left descending coronary artery as previously described by Kosutova et al. [13]. Briefly, the active substance butorphanol was administered as an analgesic at a dose of 2 mg/kg s.c. + 2 mg/kg meloxicam with 5 mL of saline + 5% glucose s.c. before surgery. The rats were intubated and ventilated with a pressure‐controlled rodent respirator (70 strokes/min) following anaesthetic treatment (i.p. with 30 mg/kg titelamine‐zolazepan). Subsequently, the rats were placed on an electric heating pad to maintain a constant body temperature at 37°C. The fifth intercostal gap was used for left lateral thoracotomy. After the pericardium was opened, the left coronary artery was identified and subsequently reversibly ligated 2 mm from the apex using silk 5‐0; C‐2 suture thread (Ethicon, San Lorenzo, USA). When ischemia was successful, the colour of the myocardial tissue changed from red to light pink. Moreover, MI was confirmed using electrocardiography (ECG). ECG was obtained from 4 needle electrodes subcutaneously inserted in the standard left—right axillar and groin sites, the rat being in the supine position (Rodent Surgical Monitor+, Indus Instruments, Webster, Texas, USA). Ligation was released 20 min after MI was induced. The surgical wound was closed in layers using Vicryl 4‐0; SH‐1 and Prolene 8‐0; CC (Ethicon, San Lorenzo, USA). The sham‐operated animals underwent the same process without ligation. Seven days after surgery, the animals were exposed to carbon dioxide vapours and after anaesthesia, exsanguination was performed.

### 2.4 Total NOS Activity

Total NOS activity was determined in 20% of tissue homogenates from the infarct zone, injury zone and nonischemic zone of the myocardium by measuring the formation of [^3^H]‐l‐citrulline from [^3^H]‐l‐arginine (MP Biochemicals, CA, USA). Fifty microliters of homogenates were incubated with a reaction mixture (10 mM NADPH, 0.5 M Tris, pH 7.4, 20 mM CaCl_2_, 100 μM l‐arginine, 1 mg/mL calmodulin, 1 mM FAD/FMN 1:1, [^3^H]‐l‐arginine and 50 mM TH_4_) in a total volume of 50 μL per sample for 30 min at 37°C. Thereafter, 1 mL of stop buffer (0.02 M HEPES pH 5.5, 2 mM EDTA, 2 mM EGTA and 1 mM l‐citrulline) was added to stop the reaction. The samples were applied to 1 mL of Dowex 50WX8, Na^+^‐form, 100–200 mesh columns together with 1 mL of distilled water. Then, 4 mL of Sigma‐FluorTM High Performance LSC Cocktail was added to 250 μL of the leaked samples. [^3^H]‐l‐Citrulline was measured with a Tri‐Carb 2910TR liquid scintillation analyser (PerkinElmer, Waltham, MA, USA). NOS activity is expressed as pkat/g protein.

### 2.5 Western Blot Analysis

Tissue samples were collected from three different parts of the myocardium (the infarct zone, injured zone and nonischemic zone). Each tissue sample (~50–100 mg) was immediately placed on ice and homogenised in ice‐cold lysis buffer containing 0.05 mM Tris (pH 7.4) supplemented with a protease inhibitor cocktail (to prevent proteolytic degradation). Homogenisation was performed using a mechanical homogeniser until complete tissue disruption, ensuring maximal release of cellular proteins. The homogenates were then centrifuged at 12,000 *g* for 15 min at 4°C to remove cellular debris. The resulting supernatants, containing the soluble protein fraction, were carefully collected and total protein concentrations were determined using the Lowry protein assay with bovine serum albumin (BSA) as the standard. Protein extracts were aliquoted and stored at −80°C until further use to avoid repeated freeze–thaw cycles. Equal amounts of protein were then separated by 12% SDS–polyacrylamide gel electrophoresis (SDS–PAGE) and transferred onto nitrocellulose membranes for immunoblotting. To prevent nonspecific binding, membranes were blocked with 5% nonfat milk in Tris‐buffered saline (TBS; pH 7.6) containing 0.1% Tween‐20 (TBS‐T) for 1 h at room temperature. After blocking, membranes were incubated overnight at 4°C with the following primary antibodies: polyclonal rabbit anti‐endothelial nitric oxide synthase (eNOS), polyclonal rabbit anti‐TLR4 (both 1:1000, Abcam, Cambridge, UK), polyclonal rabbit anti‐inducible nitric oxide synthase (iNOS, 1:1000, Bio‐Rad, Hercules, CA, USA), monoclonal mouse anti‐NFκB (1:1000, Cell Signalling, Danvers, MA, USA) and polyclonal rabbit anti‐GAPDH (1:5000, Abcam, Cambridge, UK) as a loading control. Following primary antibody incubation, membranes were washed and incubated at room temperature for 2 h with species‐specific secondary antibodies conjugated to horseradish peroxidase: goat anti‐rabbit (1:5000, Abcam, Cambridge, UK) or goat anti‐mouse (1:3000, Cell Signalling, Danvers, MA, USA). Protein bands were visualised using an enhanced chemiluminescence (ECL) detection system (Bio‐Rad, Hercules, CA, USA). Signal intensity was quantified using the ChemiDoc Touch Imaging System (Image Lab Touch software, version 5.2, Bio‐Rad, Hercules, CA, USA) and normalised to GAPDH to account for variations in protein loading.

### 2.6 Measurement of Collagen Content

The amount of collagen in tissue was quantified by measuring hydroxyproline (hyp), an amino acid that is a major component of collagen. To determine the amount of hyp in the left ventricle, we used a modified method according to Reddy and Enwemeka [15]. Briefly, we used the same homogenate as used for measurement of NOS activity. 25 μL of sample was placed into a test tube and subjected to hydrolysis by 50 μL of 2 M NaOH. The samples were then heated to 110°C for 60 min, which breaks down the tissue and converts the collagen's hydroxyproline into a free form that can be measured. After hydrolysis, the tissue samples were neutralised with 450 μL of chloramine‐T for 25 min at room temperature. Following chloramine‐T treatment, 500 μL of p‐dimethylaminobenzaldehyde (DMAB) was added to the sample. Incubation lasted 20 min at 65°C. The absorbance of the resulting solution was measured at 550 nm using a spectrophotometer (NanoDrop One, Thermo Fisher, Waltham, MA, USA). The collagen content in the tissue samples was calculated using a hydroxyproline standard calibration curve converted to the amount of protein in the homogenate. The samples were repeated in triplicate to ensure consistency in results. It is assumed that 12.5% of collagen is composed of hydroxyproline. A standard curve is prepared by creating a series of hydroxyproline standards with known concentrations.

### 2.7 Measurements of Conjugated Diene Content

The concentration of conjugated dienes was measured in lipid extracts of the left ventricle, adipose tissue and liver. The samples were homogenised in 15 mmol/dm^3^ EDTA containing 4% NaCl. Lipids were extracted using a 1:1 chloroform‐methanol mixture. Chloroform was evaporated in a N_2_ atmosphere and then cyclohexane (PanReac AppliChem, Darmstadt, Germany) was added. The conjugated diene concentrations were determined by a spectrophotometer (NanoDrop One, Thermo Fisher, Waltham, MA, USA) at *λ* = 233 nm. The concentrations were calculated using the extinction coefficient *ε* = 29,000 L/mol/cm and are expressed as nmol per g tissue.

### 2.8 Statistical Analysis

All the data were analysed by two‐way analysis of variance (ANOVA) followed by the Bonferroni post hoc correction. The data are expressed as the mean ± SEM. The level of statistical significance was set as *p* < 0.05. The data were analysed with STATISTICA 10.

## 3 Results

### 3.1 Somatic Parameters, Blood Pressure and Cytokine Levels

The average daily food intake was 23.82 ± 0.63 g per day/rat in the control group and 17.46 ± 0.19 g in the HFD group (*p* < 0.01). In both groups, after the surgical procedure, we observed a decrease in food intake (20.24 ± 0.27 g in control + MI vs. 14.78 ± 0.42 g in HFD + MI; *p* < 0.01). This decrease was also noted between the sham‐operated and infarct groups within the same diet. There was a significant difference in this parameter during the whole treatment period between the control group and the group fed an HFD before and after MI (*p* < 0.01). Body weight, heart weight and kidney weight did not differ between the groups after coronary ligation. There was a significant increase in the weight of retroperitoneal fat in the sham‐operated animals fed a high‐fat diet compared to the sham‐operated animals fed a control diet (*p* < 0.01) and among the infarcted groups fed a high‐fat and control diet (*p* < 0.01). Moreover, there was a significant increase in the plasma levels of triacylglycerol (TAG) and glucose level in both HFD groups, as expected (Table 2).

**TABLE 2:** General parameters of experimental animals.

Parameter	Control + Sham	Control + MI	HFD + Sham	HFD + MI
BW (g)	181.50 ± 4.62	181.17 ± 4.73	177.83 ± 5.11	194.83 ± 5.87
HW (g)	0.84 ± 0.13	0.91 ± 0.02	0.83 ± 0.05	0.84 ± 0.07
KW (g)	0.72 ± 0.06	0.73 ± 0.03	0.71 ± 0.02	0.72 ± 0.03
TL (mm)	37.33 ± 1.38	39.50 ± 1.50	38.17 ± 0.91	40.58 ± 1.20
RFW (g)	1.15 ± 0.11	1.36 ± 0.07	2.85 ± 0.03***	2.90 ± 0.22^###^
HW/BW (g) × 10^−3^	5 ± 0.2	5 ± 0.2	5 ± 0.2	4 ± 0.2
HW/TL (g/mm)	0.023 ± 0.1 × 10^−2^	0.023 ± 0.8 × 10^−3^	0.022 ± 0.1 × 10^−2^	0.021 ± 0.1 × 10^−2^
TAG (mmol/L)	1.21 ± 0.03	1.15 ± 0.07	2.58 ± 0.13**	2.72 ± 0.19^##^
CHOL (mmol/L)	1.25 ± 0.11	1.19 ± 0.07	1.48 ± 0.09*	1.52 ± 0.^##^
HDL (mmol/L)	0.48 ± 0.02	0.46 ± 0.06	0.40 ± 0.05**	0.41 ± 0.07^#^
GLU (mmol/L)	5.2 ± 0.09	4.9 ± 0.12	7.1 ± 0.**	6.8 ± 0.^##^
FI (g/day)	23.82 ± 0.63	20.24 ± 0.27	17.46 ± 0.19**	14.78 ± 0.42^##^

*Note:* Somatic parameters of the sham‐operated WKY rats fed a control diet (control + sham), WKY rats fed a control diet with experimentally induced myocardial infarction (control + MI), sham‐operated WKY rats fed a high‐fat diet (HFD + sham) and WKY rats fed a high‐fat diet with experimentally induced myocardial infarction (HFD + MI). The data are the means ± SEMs. * *p* < 0.05; ** *p* < 0.01; *** *p* < 0.001 vs. control + sham; # *p* < 0.05; ## *p* < 0.01; ### *p* < 0.001 vs. HFD + sham; *n* = 7−8. The data are the means ± SEMs.

Abbreviations: BW, body weight; CHOL, total cholesterol; FI, food intake; GLU, glucose; HDL, high density lipoprotein; HW, heart weight; HW/BW, ratio of heart weight to body weight; HW/TL, ratio of heart weight to tibia length; KW, kidney weight; RFW, retroperitoneal fat weight; TAG, triacylglycerol; TL, tibia length.

No significant changes were detected in blood pressure between the groups before (week 12 of age) and after experimentally induced myocardial infarction (week 13 of age). However, HFD consumption significantly increased blood pressure (*p* < 0.05) after surgery in both the sham‐operated and infarcted animals (*p* < 0.01; Table 3).

**TABLE 3:** Blood pressure before and after myocardial infarction.

	Week 12 (mmHg)	Week 13 (mmHg)
Control + Sham	119 ± 1	116 ± 2
Control + MI	114 ± 2	116 ± 1
HFD + Sham	122 ± 1	122 ± 1*
HFD + MI	119 ± 2	122 ± 1^##^

*Note:* Blood pressure of the sham‐operated WKY rats fed a control diet (control + sham), WKY rats fed a control diet with experimentally induced myocardial infarction (control + MI), sham‐operated WKY rats fed a high‐fat diet (HFD + sham) and WKY rats fed a high‐fat diet with experimentally induced myocardial infarction (HFD + MI). Week 12—before surgery; week 13–7 days after surgery; *n* = 7–8. The data are the means ± SEMs.

* *p* < 0.05 versus control + sham.

^##^ *p* < 0.01 versus control + MI.

We evaluated plasma cytokine levels before and after MI. As shown in Table 4, the levels of TNF‐α and IL‐6 were higher in the MI group than in the control group by 119.7% and 98.7%, respectively (*p* < 0.001). Similarly, the concentrations of TNF‐α and IL‐6 were 76.3% and 70.3% greater, respectively, in the HFD groups than in the control diet groups (*p* < 0.001). Moreover, the administration of an HFD increased TNF‐α and IL‐6 levels by 56.8% and 27%, respectively, in the sham‐operated rats (HFD + sham) versus the control + sham rats (*p* < 0.001) and by 25.9% and 8.9%, respectively, in the infarcted animals (HFD + MI) vs. the control + MI animals (*p* < 0.001). Furthermore, troponin levels were elevated after myocardial infarction in both groups, regardless of whether a control or high‐fat diet was administered (*p* < 0.001). Moreover, we evaluated cardiac antioxidant enzyme activity across the experimental groups. As shown in Table 4, catalase activity was reduced in the MI group by 56% compared with the sham control animals (*p* < 0.001). Likewise, catalase activity decreased by 27% in the HFD group and by 64% in the HFD + MI group relative to the sham controls (*p* < 0.01). A similar pattern was observed for SOD activity: MI reduced SOD activity by 39%, HFD by 30% and the combination of HFD and MI by 49% compared with the sham control group (*p* < 0.001; Table 4).

**TABLE 4:** Inflammatory, cardiac and antioxidant markers.

Parameter	Control + Sham	Control + MI	HFD + Sham	HFD + MI
TNF‐α (pg/mL)	13.24 ± 0.27	29.00 ± 0.40***	20.68 ± 0.58***	36.53 ± 0.45^###^
IL‐6 (pg/mL)	23.33 ± 0.52	46.30 ± 0.59***	29.58 ± 0.53***	50.43 ± 0.28^###^
Troponin (pg/mL)	4.07 ± 0.63	21.81 ± 2.93***	2.64 ± 0.26	28.87 ± 3.13^###^
Catalase (U/mL)	1.60 ± 0.20	0.71 ± 0.15***	1.17 ± 0.11**	0.58 ± 0.20^##^
SOD (U/mL)	5.50 ± 0.12	3.36 ± 0.07***	3.85 ± 0.03***	2.85 ± 0.12^###^

*Note:* Cytokine and troponin levels, catalase and superoxide dismutase activities of the sham‐operated WKY rats fed a control diet (control + sham), WKY rats fed a control diet with experimentally induced myocardial infarction (control + MI), sham‐operated WKY rats fed a high‐fat diet (HFD + sham) and WKY rats fed a high‐fat diet with experimentally induced myocardial infarction (HFD + MI). Tumour necrosis factor‐α (TNF‐α); interleukin‐6 (IL‐6); catalase (Cat); superoxide dismutase (SOD); *n* = 7–8. The data are the means ± SEMs.

** *p* < 0.01.

*** *p* < 0.001 versus control + sham.

^##^ *p* < 0.01.

^###^ *p* < 0.001 versus HFD + sham.

### 3.2 Total Activity of NOS

**FIGURE 1:**
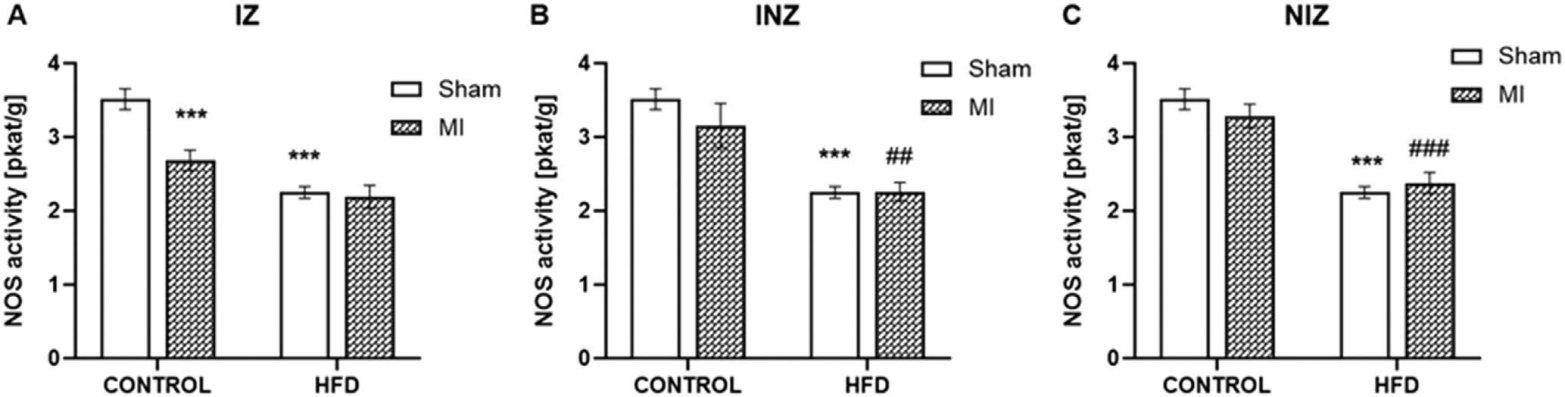
Nitric oxide synthase activity. Total nitric oxide synthase (NOS) activity in the infarcted zone (IZ; A), injured zone (INZ; B) and nonischemic zone (NIZ; C) of the myocardium after the induction of myocardial infarction and administration of a high‐fat diet (HFD) in the sham‐operated WKY rats fed a control diet (control + sham), WKY rats fed a control diet with experimentally induced myocardial infarction (control + MI), sham‐operated WKY rats fed a high‐fat diet (HFD + sham) and WKY rats fed a high‐fat diet with experimentally induced myocardial infarction (HFD + MI). ***p < 0.001 versus control + sham; ^##^ *p* < 0.01; ^###^ *p* < 0.001 versus control + MI; *n* = 7–8. The data are the means ± SEMs.

Myocardial infarction significantly reduced total nitric oxide synthase (NOS) activity within the infarct zone (IZ) compared to that in the sham‐operated hearts (control + sham) (*p* < 0.001). Interestingly, NOS activity remained unchanged in the injured zone (INZ) and nonischemic zone (NIZ) between the infarcted and control groups. Furthermore, HFD itself led to a significant decrease in NOS activity across all myocardial zones in the HFD + sham compared to the controls (control + sham) (*p* < 0.001). Moreover, the combination of an HFD and MI further decreased NOS activity. This effect was observed in the injured zone (INZ) compared to that in the controls with MI (*p* < 0.01) and even in the nonischemic zone (NIZ) (*p* < 0.001) (Figure 1A–C).

### 3.3 Protein Expression

**FIGURE 2:**
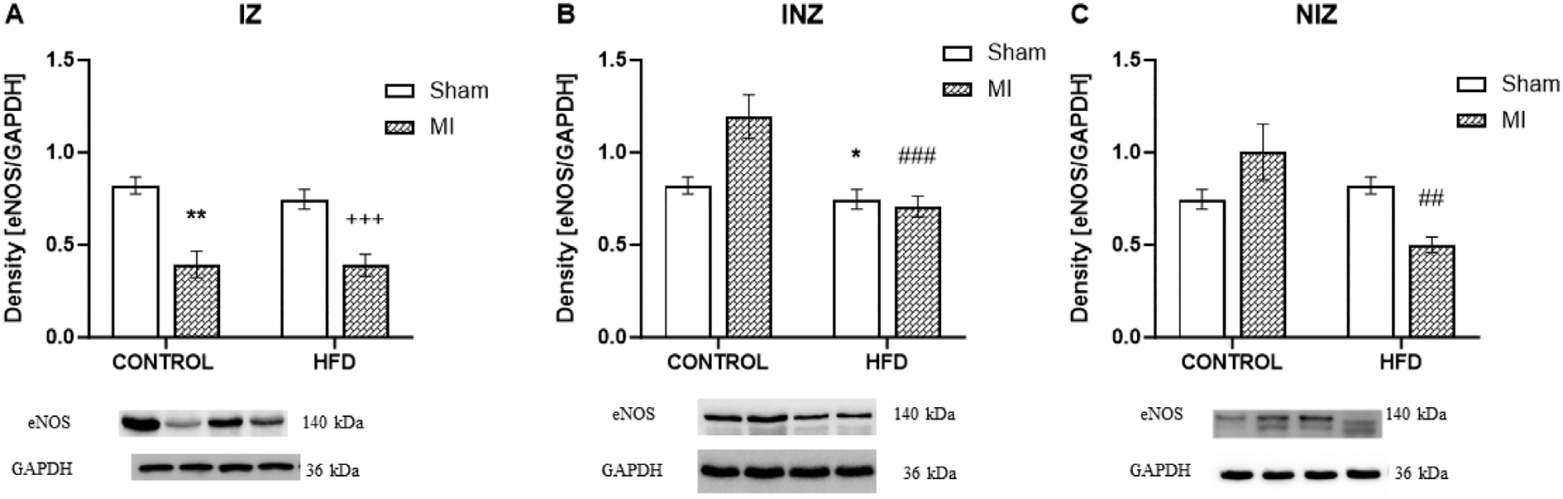
Expression of endothelial nitric oxide synthase. Expression of endothelial nitric oxide synthase (eNOS) in the infarcted zone (IZ; A), injured zone (INZ; B) and nonischemic zone (NIZ; C) of the myocardium after induction of myocardial infarction and administration of a high‐fat diet in the sham‐operated WKY rats fed a control diet (control + sham), WKY rats fed a control diet with experimentally induced myocardial infarction (control + MI), sham‐operated WKY rats fed a high‐fat diet (HFD + sham) and WKY rats fed a high‐fat diet with experimentally induced myocardial infarction (HFD + MI). *p < 0.05; **p < 0.01 versus control + sham; ^++^ *p* < 0.01 versus HFD + sham; ^##^ *p* < 0.01; ^###^ *p* < 0.001 versus control + MI; *n* = 7–8. The data are the means ± SEMs.

We determined the protein expression of eNOS, iNOS, NFkB and TLR4 in the infarct zone, injury zone and nonischemic zone of the heart with or without an HFD. The protein expression of eNOS, a beneficial enzyme, was downregulated in the IZ after MI in the control group (*p* < 0.01) and the group fed a high‐fat diet (*p* < 0.001). This finding indicates impaired nitric oxide production in the damaged area. Interestingly, eNOS expression remained unchanged in the INZ and NIZ of the control animals after MI. However, an HFD appeared to have a detrimental effect even in these areas. In the INZ, eNOS expression was downregulated in both the infarcted and sham HFD groups (*p* < 0.001 and *p* < 0.05, respectively). Similarly, the NIZ showed a significant decrease in eNOS expression in the infarcted group but not in the HFD group (*p* < 0.01; Figure 2A–C).

**FIGURE 3:**
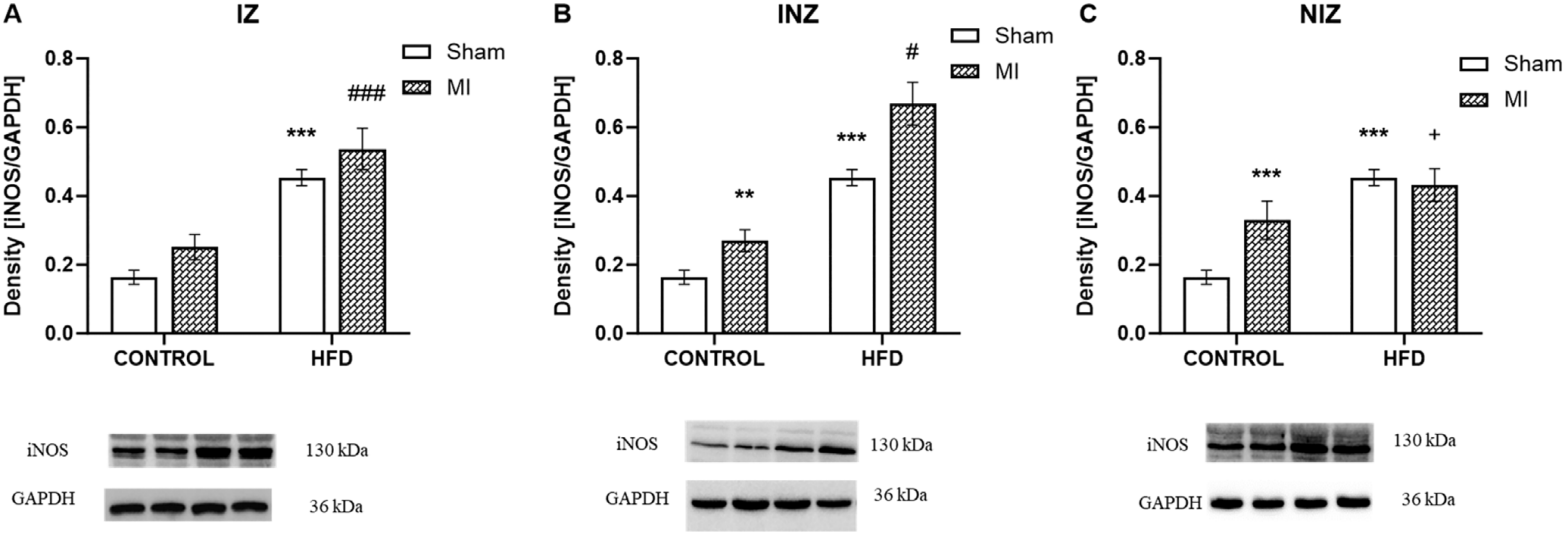
Expression of inducible nitric oxide synthase. Expression of inducible nitric oxide synthase (iNOS) in the infarcted zone (IZ; A), injured zone (INZ; B) and nonischemic zone (NIZ; C) of the myocardium after the induction of myocardial infarction and administration of a high‐fat diet in the sham‐operated WKY rats fed a control diet (control + sham), WKY rats fed a control diet with experimentally induced myocardial infarction (control + MI), sham‐operated WKY rats fed a high‐fat diet (HFD + sham) and WKY rats fed a high‐fat diet with experimentally induced myocardial infarction (HFD + MI). **p < 0.01; ***p < 0.001 versus control + sham; ^#^ *p* < 0.05; ^###^ *p* < 0.001 versus control + MI; + *p* < 0.05 versus HFD + sham; *n* = 7–8. The data are the means ± SEMs.

Unlike that of eNOS, the expression of iNOS, an enzyme with complex roles, did not significantly change in the infarct zone after MI; however, it tended to increase between the infarcted and sham‐operated animals fed a control diet. Significantly increased iNOS expression in the INZ (*p* < 0.01) and in the NIZ (*p* < 0.001) was found in the infarcted animals fed a control diet compared to the sham‐operated animals. Interestingly, a high‐fat diet seemed to have opposed effects on iNOS expression in the NIZ compared to the control diet. In the HFD‐fed animals, iNOS expression in the NIZ was decreased in the infarct group compared to the sham group (*p* < 0.05). The effect of diet on the IZ, INZ and NIZ caused a significant increase in iNOS expression in the animals fed a high‐fat diet compared to that in the animals fed a control diet (all comparisons *p* < 0.05 or *p* < 0.001; Figure 3A–C).

**FIGURE 4:**
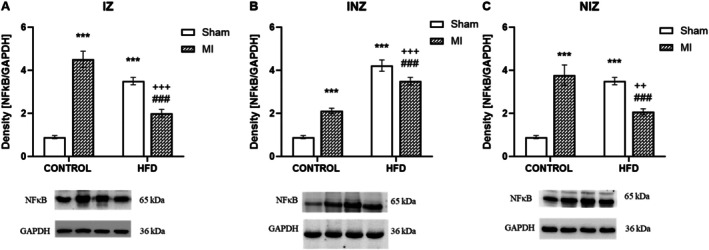
Expression of nuclear factor kappa B. Expression of nuclear factor kappa B (NFκB) in the infarcted zone (IZ; A), injured zone (INZ; B) and nonischemic zone (NIZ; C) of the myocardium after induction of myocardial infarction and administration of a high‐fat diet in the sham‐operated WKY rats fed a control diet (control + sham), WKY rats fed a control diet with experimentally induced myocardial infarction (control + MI), sham‐operated WKY rats fed a high‐fat diet (HFD + sham) and WKY rats fed a high‐fat diet with experimentally induced myocardial infarction (HFD + MI). ***p < 0.001 versus control + sham; ^###^ *p* < 0.001 versus control + MI; ^++^ *p* < 0.01; ^+++^ *p* < 0.001 versus HFD + sham; *n* = 7–8. The data are the means ± SEMs.

Another inflammatory signalling molecule, NFκB, showed interplay between MI and an HFD. In the control animals, MI significantly increased NFκB expression across all investigated heart zones compared to that in the sham animals (*p* < 0.001). Interestingly, an HFD seemed to have opposite effects on NFκB expression depending on MI. In the infarcted animals fed an HFD, NFκB expression in the IZ and NIZ was decreased compared to that in the control group (*p* < 0.001). However, in the INZ, HFD reversed this trend, leading to a significant increase in NFκB expression in the infarcted animals compared to the controls (*p* < 0.001). NFκB expression in the IZ and NIZ was significantly lower in the infarcted animals fed a high‐fat diet than in the infarcted animals fed a control diet (*p* < 0.001). In the INZ, in contrast, NFκB expression was significantly greater in the infarcted animals fed a high‐fat diet than in the controls (*p* < 0.001; Figure 4A–C).

**FIGURE 5:**
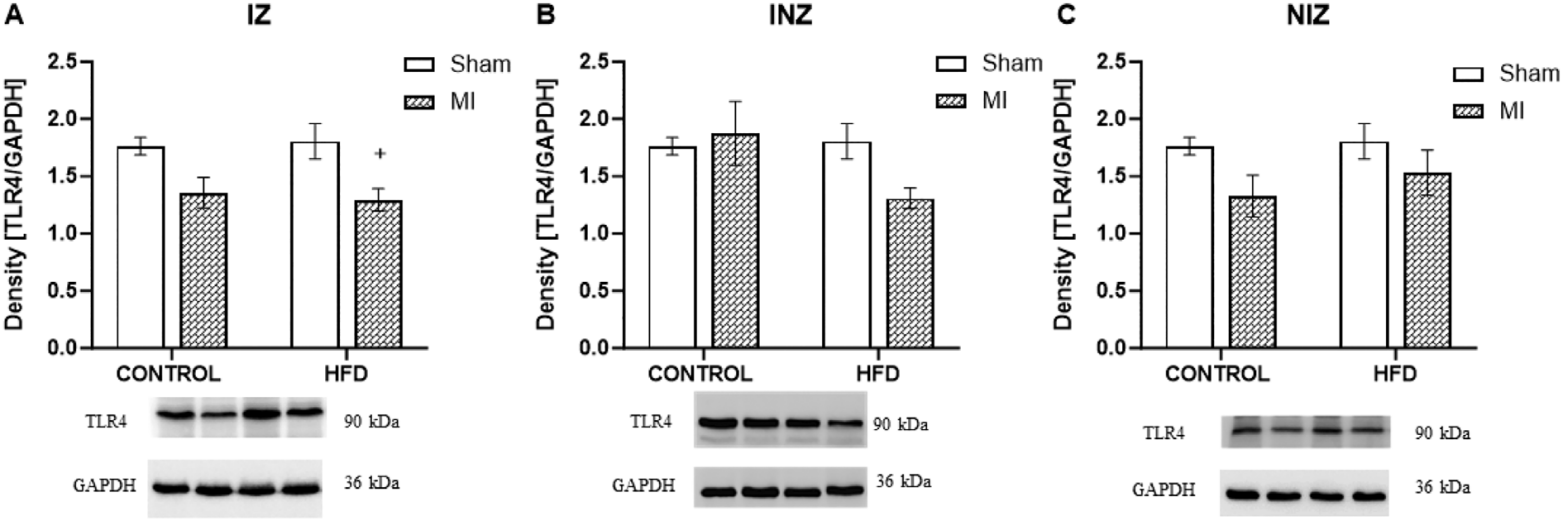
Expression of toll‐like receptor. Expression of Toll‐like receptor 4 (TLR4) in the infarcted zone (IZ; A), injured zone (INZ; B) and nonischemic zone (NIZ; C) of the myocardium after induction of myocardial infarction and administration of a high‐fat diet in the sham‐operated WKY rats fed a control diet (control + sham), WKY rats fed a control diet with experimentally induced myocardial infarction (control + MI), sham‐operated WKY rats fed a high‐fat diet (HFD + sham) and WKY rats fed a high‐fat diet with experimentally induced myocardial infarction (HFD + MI). ^+^ *p* < 0.05 versus HFD + sham; *n* = 7–8. The data are the means ± SEMs.

In the next step, we investigated Toll‐like receptor 4, a molecule involved in the inflammatory process, as well. We observed a significant decrease in TLR4 expression in the IZ in the infarcted animals fed a high‐fat diet compared to that in the sham controls (*p* < 0.05). There were no differences among the other groups or in other myocardial zones (Figure 5A–C).

### 3.4 Collagen Content

**FIGURE 6:**
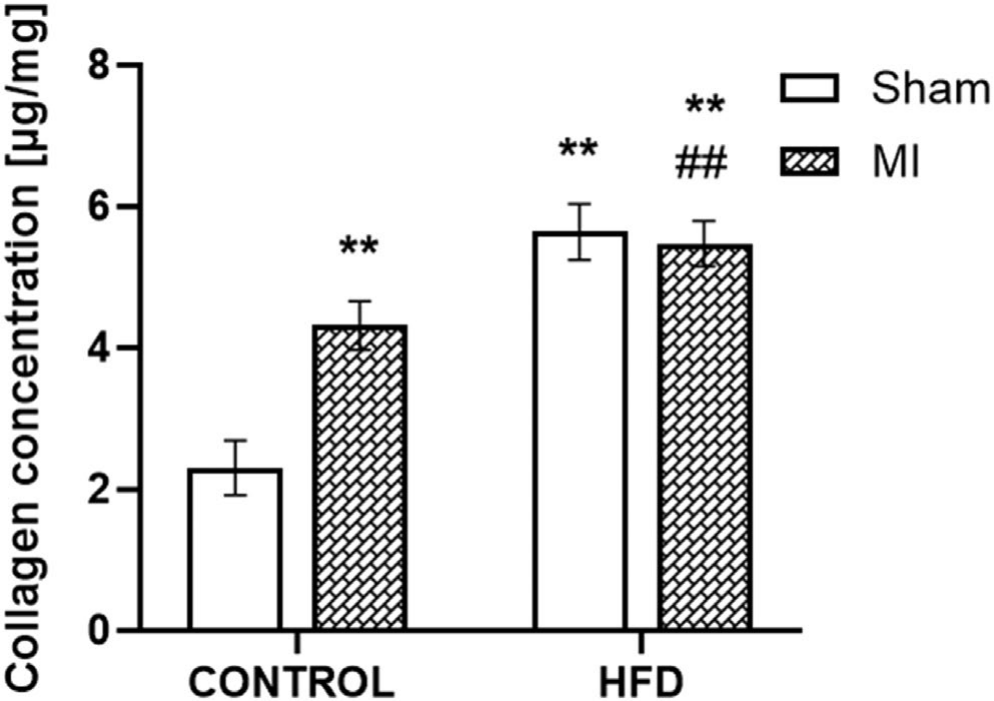
Collagen content. Collagen content in the left ventricle of the heart in the sham‐operated WKY rats fed a control diet (control + sham), WKY rats fed a control diet with experimentally induced myocardial infarction (control + MI), sham‐operated WKY rats fed a high‐fat diet (HFD + sham) and WKY rats fed a high‐fat diet with experimentally induced myocardial infarction (HFD + MI). **p < 0.01 versus control + sham; ^##^ *p* < 0.01 versus control + MI; *n* = 8. The data are the means ± SEMs.

Considering that myocardial infarction induces heart remodelling, we assessed the extent of changes in collagen content. Additionally, we investigated whether the administration of a high‐fat diet only influences collagen levels in the myocardium, irrespective of surgical intervention. A significant increase in collagen levels was observed in the group following myocardial infarction fed a normal diet compared to the control + sham group (4.31 ± 0.34 μg/mg vs. 2.31 ± 0.39 μg/mg; *p* < 0.05). Moreover, a significant increase in collagen levels was detected in both MI and sham HFD groups compared with controls (5.64 ± 0.39 and 5.48 ± 0.32 μg/mg, respectively; *p* < 0.01; Figure 6).

### 3.5 Conjugated Diene Concentrations

**FIGURE 7:**
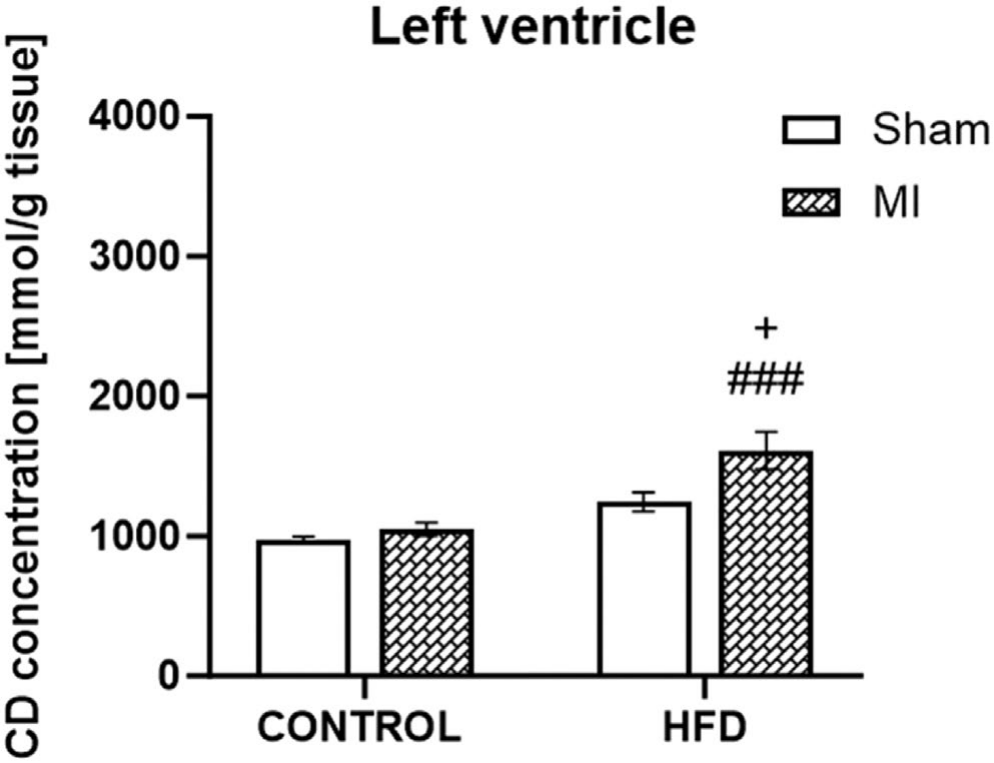
Concentration of conjugated dienes. Concentration of conjugated dienes in the left ventricle of the heart in the sham‐operated WKY rats fed a control diet (control + sham), WKY rats fed a control diet with experimentally induced myocardial infarction (control + MI), sham‐operated WKY rats fed a high‐fat diet (HFD + sham) and WKY rats fed a high‐fat diet with experimentally induced myocardial infarction (HFD + MI). ^###^ *p* < 0.001 versus control + MI; ^+^ *p* < 0.05; ^++^ *p* < 0.01 versus HFD + sham; *p < 0.05; ***p < 0.001 versus control + sham; *n* = 8. The data are the means ± SEMs.

The concentration of conjugated dienes, a marker of oxidative damage, was measured in the left ventricle of the heart. There was some effect of the induction of MI in all investigated tissues. In the left ventricle, a significantly greater concentration of conjugated dienes was detected in the HFD + MI group than in the HFD + sham group and in the control + MI group (*p* < 0.05; *p* < 0.001; Figure 7).

## 4 Discussion

Cardiovascular disease (CVD) remains the leading cause of death, and obesity and inflammation are risk factors for CVD development and progression. This strong association highlights the critical need for understanding the mechanisms by which excess body fat contributes to CVD. Our experimental approach involved feeding a control diet and high‐fat diet with a composition equivalent to that of today's fast food. We aimed to analyse the biochemical changes after MI in WKY rats with a focus on ongoing inflammatory processes in different areas of the heart and oxidative stress resulting from the formation of ROS.

In our study, we did not observe significant differences in blood pressure after MI. In contrast to our results, several other studies have shown a significant reduction in blood pressure after MI [16, 17]. We assume a smaller extent of damage due to reperfusion, which does not affect either the contractility or the cardiac output, on which blood pressure depends. In contrast, after HFD feeding, we observed a significant increase in blood pressure compared to that of the animals fed the control diet. A high‐fat diet can lead to increased activity of the sympathetic nervous system, which can cause constriction of blood vessels and thus lead to increased blood pressure. Administration of HFD may affect the structure of the lipid bilayer of the cell membrane due to intracellular lipid accumulation and thus significantly affect the function of the membranes and organs and lead to contractile dysfunction by inducing loss of caveolin‐3 from the T‐tubule system of the membrane [18].

A high‐fat diet resulted in a significant increase in visceral fat mass without an increase in total body weight, which is consistent with the results reported by other authors [19, 20]. A high‐fat diet contains a large amount of energy that animals are not able to use, and it is stored in adipose tissue. The accumulation of adipose tissue, particularly visceral fat, was linked to an increase in triglyceride levels in the bloodstream in both HFD groups. Our previous study suggests that adipose tissue is not merely a passive fat store but functions as an active endocrine organ that modulates systemic inflammation, endothelial function and myocardial remodelling [21, 22].

In the infarcted myocardium, ongoing inflammation plays a central role in post‐injury remodelling. Following coronary occlusion, the ischemic tissue releases distress signals via DAMPs, which serve as ligands for pattern recognition receptors such as TLR4. Some studies have shown that inflammatory cytokines are involved in myocardial remodelling [23]. This triggers an inflammatory cascade mediated by cytokines including TNF‐α and IL‐6, which we found to be elevated both after MI and in animals fed a high‐fat diet. Adverse effects of MI on cytokine levels were also described by Elseweidy et al. [24] for isoprenalin and Karimkhani et al. [25] for an isoproterenol‐HCl‐induced experimental rat model. Duan et al. [26] reported a significant increase in cytokine levels 1 week after MI in human patients. A high‐fat diet also affected cytokine levels in plasma. Importantly, this inflammatory surge was not limited to infarcted tissue, implying systemic immune activation. Shirvani et al. [27] described increased expression of TNF‐α and IL‐6 in the skeletal muscle of Wistar rats after they were fed a high‐fat diet for 8 weeks. Our results are also consistent with the study by Chen et al. [28], in which after 4 weeks of a high‐fat diet, Sprague–Dawley rats showed increased serum levels of TNF‐α and IL‐6. In addition, a high‐fat diet has been shown to affect the development of insulin resistance. In the case of insulin resistance, lipolysis occurs with the release of fatty acids that activate NFκB; therefore, TNF‐α and IL‐6 are expressed.

Nitric oxide is a versatile signalling molecule with crucial roles in cardiovascular health. In the context of myocardial infarction, NO plays a complex dual role, balancing both protective and potentially detrimental effects depending on the concentration, cellular source and redox environment. Under physiological conditions, low concentrations of NO produced by eNOS and nNOS maintain vascular tone and inhibit leukocyte adhesion, platelet aggregation and oxidative stress. NO suppresses the expression of adhesion molecules such as P‐selectin, CD11 and CD18, thereby preventing excessive neutrophil and monocyte recruitment. It also delays endothelial apoptosis and mitigates inflammatory signalling. However, under oxidative stress, NO may participate in lipid peroxidation and endothelial injury. Superoxide reacts rapidly with NO to generate peroxynitrite, which oxidises LDL and promotes monocyte recruitment and foam cell formation, whereas excess NO alone can scavenge ROS and limit oxidative damage. Thus, the overall effect depends on the redox balance between NO and superoxide production [29].

In our study, a significant decrease in total NOS activity after experimentally induced MI was observed only in the infarct zone, indicating a localised reduction in NO production and bioavailability. Although NOS activity in other zones was not altered by MI alone, it was markedly reduced in all myocardial regions following the combination of MI and a high‐fat diet. The reduction in NO bioavailability may contribute to the increased blood pressure observed in these animals. Given the essential role of NOS in maintaining cardiovascular homeostasis, these findings suggest that both MI and HFD exert detrimental effects on endothelial and myocardial function. Analysis of individual NOS isoforms revealed that MI significantly decreased eNOS expression in the infarct zone of both control and HFD‐fed animals, while its levels remained unchanged in nonischemic regions. In contrast, iNOS expression was markedly upregulated in both injured and nonischemic zones due to MI. An increasing trend was also observed in the infarct zone. These findings are consistent with our previous study [13] and with Xu et al. [30], who observed decreased eNOS expression and increased iNOS expression 8 days after MI in Sprague–Dawley rats in which MI was induced by isoproterenol.

We assume that during the early reperfusion phase, iNOS expression markedly increases as part of the inflammatory and redox response to ischemic injury. During inflammation, cytokines such as TNF‐α, IL‐1β, IFN‐γ and LPS induce iNOS expression in macrophages, neutrophils and other immune cells, leading to the production of NO at levels up to 1000‐fold higher than normal. This massive NO release is initially protective, as it promotes microbial defence and limits vascular injury, but in a highly oxidative environment it can become cytotoxic [29]. Moreover, these cytokines stimulate the NFκB and STAT1 pathways, which strongly upregulate transcription of the NOS2 gene encoding iNOS. The resulting NO deficiency eliminates feedback inhibition on NFκB, further amplifying iNOS induction [31]. We therefore propose that early iNOS upregulation represents a compensatory mechanism to restore NO bioavailability under conditions of eNOS dysfunction. However, under oxidative conditions, excessive iNOS‐derived NO rapidly reacts with superoxide to form peroxynitrite, shifting its function from protective to cytotoxic [32]. The apparent paradox of markedly increased iNOS expression despite reduced total NOS activity can thus be attributed to oxidative uncoupling of both iNOS and eNOS, which promotes superoxide and peroxynitrite generation instead of bioactive NO. This imbalance between enzymatic expression and functional activity contributes to endothelial dysfunction and aggravates myocardial damage after infarction. Our results are consistent with findings of Momot et al. [33], who reported similar alterations in post‐MI heart failure models and after HFD administration, where iNOS upregulation was accompanied by downregulation of eNOS and nNOS. These data support our hypothesis that oxidative and metabolic disturbances, particularly those induced by HFD, promote NOS uncoupling and the formation of reactive nitrogen species at the expense of protective NO signalling. Consistently, HFD markedly reduced eNOS expression in both injured and nonischemic zones, while iNOS expression increased throughout the heart.

The mechanisms underlying these effects appear multifactorial. Elevated TNF‐α levels in obesity downregulate eNOS mRNA stability [34, 35]. Moreover, reduced tetrahydrobiopterin (BH_4_) availability and its oxidative conversion to dihydrobiopterin in obese rodents promote eNOS uncoupling and superoxide production. In this context, macrophage‐derived iNOS becomes a predominant source of NO and reactive nitrogen species, further amplifying nitrosative stress. Consistent with this mechanism, both Li et al. [36] and Wilmes et al. [11] reported iNOS activation and cardiomyocyte damage in the early post‐MI stages in rats and humans.

NFκB itself is a central transcription factor involved not only in inflammatory signalling but also in regulating cell survival, immune responses and apoptosis. In our study, NFκB expression mirrored iNOS expression across myocardial zones in animals fed the control diet. Its activation following MI represents a typical pathophysiological response to injury: NFκB translocates from the cytoplasm to the nucleus, binds to gene promoters and triggers transcription of proinflammatory mediators [37, 38]. Increased NFκB activation during ischemia and reperfusion has been observed in both mice [39, 40] and rats [41, 42] exposed to a HFD, and we confirmed this effect in our model as well. This likely reflects systemic inflammation associated with HFD‐induced changes in macrophage phenotype from anti‐ to proinflammatory. This finding may be related to ongoing inflammation, a change in the phenotype of macrophages from anti‐inflammatory to proinflammatory in hypertrophic adipose tissue, which influences the development of systemic inflammation. The release of proinflammatory cytokines may activate NFκB, thereby inducing the expression of other proinflammatory cytokines.

Interestingly, however, the HFD had an opposite effect in infarcted animals, where NFκB expression was reduced across all myocardial zones. This finding may relate to the so‐called ‘obesity paradox,’ suggesting that despite obesity being a major risk factor for cardiovascular disease, obese patients experiencing MI may have a better prognosis than lean individuals. Chronic low‐grade inflammation associated with obesity might ‘precondition’ NFκB, rendering it less responsive to acute inflammatory activation during MI. Moreover, the combination of HFD and MI, together with elevated iNOS expression, could increase the inhibitory IκB subunit or cause nitrosylation of the NFκB p50 subunit, preventing its nuclear translocation and activation.

Together, these findings indicate that both MI and HFD disturb the fine balance between redox and inflammatory signalling that regulates NO homeostasis. NOS uncoupling, excessive iNOS‐derived NO and ROS overproduction converge to produce peroxynitrite, which oxidises BH_4_ and heme groups, nitrates catalytic residues and irreversibly impairs NOS function. Additional oxidative modifications such as S‐glutathionylation of eNOS further diminish its catalytic efficiency [43]. Both substrate and cofactor limitations reduced l‐arginine availability and restrict NO synthesis despite high iNOS expression [29]. Overall, our data support a model in which myocardial infarction and high‐fat diet synergistically impair NO signalling through redox‐dependent NOS uncoupling. This process enhances oxidative and nitrosative stress, drives endothelial dysfunction and exacerbates myocardial injury. Restoring NOS coupling and maintaining adequate antioxidant and cofactor availability may therefore represent key therapeutic targets to preserve NO bioactivity and mitigate post‐infarction remodelling.

TLRs are type I transmembrane receptors with endogenous and exogenous ligand binding abilities that stimulate innate and adaptive immune responses by inducing the immune and inflammatory cytokines IL‐6, TNF‐α and other genes. TLRs are expressed differently in immune cells and nonimmune cells, such as cardiomyocytes and endothelial cells in the heart and are involved in cardiac stress responses. TLR4 plays an important role in mediating immune cell infiltration, cytokine production and complement activation during ischemia/reperfusion [44]. Several studies have shown that TLR4 activates the expression of multiple genes encoding proinflammatory cytokines through NFκB, which plays an important role in the inflammatory response following MI or I/R injury [37, 38]. In our work, we demonstrated an increase in the concentrations of the proinflammatory cytokines TNF‐α and IL‐6 as well as an increase in the expression of NFκB. Surprisingly, there was no increase in TLR4 expression. Moreover, in the infarct zone, the expression of this molecule was decreased. In their study, Timmers et al. [45] visualised the presence of TLR4 in the infarct zone by histochemical staining, but these researchers did not detect changes by quantifying its protein expression 4 days after MI. Saturated fatty acids, particularly lauric acid and palmitic acid, were found to stimulate an inflammatory response through the TLR4 signalling pathway [46]. We assumed that the food content differed from that of saturated fatty acids and that monounsaturated and polyunsaturated acids did not affect TLR4 signal activation. In addition to the NF‐kB and TLR4 signalling pathways, several alternative cascades may contribute to myocardial infarction and high‐fat diet induced fibrosis and remodelling. The JAK/STAT pathway is a key player in inflammation and fibrosis post‐MI, activated by cytokines like IL‐6 and IFN‐γ [47]. In the presence of HFD, this pathway amplifies the fibrotic response, particularly in cardiac fibroblasts. The MAPK pathway, including p38 MAPK, ERK1/2 and JNK, is also crucial in MI‐related remodelling. HFD‐induced oxidative stress exacerbates MAPK activation, promoting fibroblast proliferation and collagen deposition. Additionally, AMPK, a regulator of cellular energy balance, plays an important role in reducing inflammation and fibrosis. A high‐fat diet reduces AMPK activity, which may exacerbate the fibrotic response [48]. These alternative signalling pathways suggest additional regulatory mechanisms in cardiac remodelling after MI, independent of the NF‐kB and TLR4 pathways.

Myocardial infarction leads to significant myocardial damage, initiating a complex healing process involving inflammation, tissue repair and fibrosis, with collagen playing a central role in tissue repair and structural support. Following MI, fibroblasts become activated and produce increased collagen to form scar tissue, a key aspect of the healing process. Our results show increased collagen content post‐MI, consistent with findings by Rios‐Navarro et al. [49] in swine models. Collagen deposition and extracellular matrix remodelling are crucial for restoring structural integrity to the heart, but excessive fibrosis can negatively impact cardiac function. This fibrotic response is triggered by inflammation, where fibroblasts proliferate and secrete collagen as part of the healing process, but excessive fibrosis can contribute to adverse remodelling [50]. The high‐fat diet further influences collagen production, as it promotes systemic inflammation and metabolic disturbances like oxidative stress, which exacerbate fibrosis. HFD contributes to pro‐inflammatory mediator production, stimulating collagen synthesis. Additionally, HFD‐induced endothelial dysfunction may further promote collagen accumulation in the myocardium, exacerbating tissue damage. Our findings suggest that a high‐fat diet can enhance collagen deposition not only after MI, and consequently potentially worsening fibrosis and heart failure. Thus, while collagen production is necessary for healing, a high‐fat diet can amplify fibrosis through inflammatory and metabolic pathways, impairing myocardial function.

We further evaluated conjugated dienes as a marker of oxidative damage in left ventricular cardiac tissue. As expected, we observed a significant increase in the concentration of conjugated dienes after MI, which is consistent with other studies. Radhiga et al. [51] showed increased conjugated dienes in the heart and plasma of Wistar rats after isoproterenol‐induced MI. Cardoso et al. [52] reported that a high‐fat diet induces oxidative stress, lipid peroxidation and tissue damage. ROS formation after MI is likely related to iNOS expression. Since we did not compare time‐dependent changes induced by MI, we cannot determine whether these changes represent a transient or sustained effect. The elevated conjugated diene levels may be linked to reperfusion injury, characterised by a burst of ROS production upon the restoration of oxygen supply. Excessive ROS generation leads to oxidative damage of cellular structures when antioxidant defences, including superoxide dismutase, catalase and glutathione, are insufficient. Reduced activity of these enzymes facilitates oxidative stress, lipid and protein oxidation and mitochondrial dysfunction within the infarcted myocardium. A high‐fat diet further exacerbates these effects by promoting ROS formation and impairing antioxidant capacity. The combined impact of MI‐induced oxidative stress and HFD‐related metabolic imbalance may amplify inflammatory signalling and fibrotic remodelling, resulting in adverse cardiac outcomes. Activation of molecular pathways such as NF‐κB and TGF‐β promotes pro‐inflammatory cytokine production and collagen deposition, increasing tissue vulnerability and hindering post‐infarction recovery. The observed increase in adipose tissue mass may reflect adipocyte hypertrophy, a condition accompanied by low‐grade inflammation, cytokine release and ROS generation. This was supported by our findings of elevated conjugated dienes in the left ventricle. We propose that the elevation of conjugated dienes in the myocardium after MI and HFD exposure, together with increased pro‐inflammatory cytokine production, may stem from NF‐κB pathway activation during the early post‐infarction phase. This activation likely enhances transcription of inflammation‐ and oxidative stress‐related genes, leading to excessive ROS generation and lipid peroxidation. Consequently, the interplay between ischemia‐induced oxidative stress and diet‐related metabolic dysregulation may sustain NF‐κB‐driven inflammatory cascades, contributing to persistent redox imbalance and membrane lipid damage in cardiac tissue.

## 5 Conclusion

A high‐fat diet is detrimental to cardiovascular health and can significantly worsen the outcome of a myocardial infarction. Our findings demonstrate that a high‐fat diet exacerbates myocardial injury after infarction by amplifying inflammation, impairing nitric oxide signalling and increasing oxidative stress. HFD‐fed animals showed sustained elevation of TNF‐α and IL‐6, reduced eNOS expression and increased iNOS levels, indicating a proinflammatory myocardial environment with decreased NO bioavailability. Furthermore, the process of lipid peroxidation was found to be enhanced, thus contributing to additional tissue damage. Overall, these results highlight that a high‐fat diet has a detrimental effect on post‐MI cardiac remodelling and repair, emphasising the importance of maintaining a healthy diet to preserve nitric oxide function and support cardiovascular recovery.

## 6 Limitation of the Study

The limitation of this study is the lack of cardiac function data. Echocardiography would have provided a more reliable assessment of cardiac function; however, at the time of the experiments, an echocardiogram was not available at the Institute and it was not feasible to transfer the animals to another facility for this procedure due to Covid‐19 restriction.

## Author Contributions

Conceptualisation: M.C. and A.B.; Methodology: K.B., A.B., J.L. and M.C.; Investigation: K.B., A.B. and M.C.; Writing: original draft: K.B. and M.C.; Review and editing: M.C.; Visualisation: K.B. and M.C.; Funding acquisition: M.C.

## Funding

This work was supported by national grant agencies Slovak Research and Development Agency (Grant APVV‐22‐0271) and Grant Agency of the Ministry of Education, Research, Development and Youth of the Slovak Republic (Grant VEGA 2/0131/24).

## Conflicts of Interest

The authors declare no conflicts of interest.

## Data Availability

The raw data supporting the conclusions of this study is available from the corresponding author upon request, without undue reservation.
